# Evaluating Soft Tissue Healing after Implant Placement Using Two Different Mouthwashes (Myrrh and Chlorhexidine Gluconate): A Randomized Control Trial

**DOI:** 10.3390/medicina58101351

**Published:** 2022-09-26

**Authors:** Wael I. Ibraheem, Afaf A. Hakami, Ahlam A. Shafei, Salehah A. Jerah, Ammar Almarghlani, Ali M. Makrami, Ashok K. Bhati

**Affiliations:** 1 Department of Preventive Dental Sciences, College of Dentistry, Jazan University, Jazan 45142, Saudi Arabia; 2 College of Dentistry, Jazan University, Jazan 45142, Saudi Arabia; 3 Department of Periodontics, Faculty of Dentistry, King Abdul Aziz University, Jeddah 22254, Saudi Arabia; 4 Oral and Maxillofacial Surgery Consultant, Prince Mohammed Bin Nasser Hospital, Jazan 82943, Saudi Arabia

**Keywords:** myrrh, chlorhexidine, dental implant, healing

## Abstract

The use of mouthwash is often recommended by dental experts for dental healing. A double-blind, randomized clinical study was conducted to evaluate the efficacy of two mouthwashes (myrrh and chlorhexidine gluconate) on postoperative pain and their effects on tissues after dental implant placement in 35 patients (18 in the myrrh group and 17 in the chlorhexidine gluconate group). Soft tissue healing was evaluated in terms of wound closure, soft tissue swelling, and the color of the gingiva at 1 week postoperative. To decrease the chances for error, only the participants who did not show preoperative symptoms of infection and those who committed to practicing better oral hygiene were included in the study. The samples were evaluated for the infiltration of inflammatory cells (using inflammatory extent and inflammatory cellularity grades), maturation of collagen (osteoblast activity), and arrangement of cells (for detecting the remodeling phase). A questionnaire pertaining to mouthwash satisfaction, the duration of postoperative pain after the procedure, the time of stoppage of bleeding at the surgical site, and any sensitivity at the surgical site was given to the patients. The Chi-square test and Mann–Whitney U-test were used to analyze the data. The difference in postoperative surgical swelling, pain, bleeding, and redness in the patients was not statistically significant between the myrrh and chlorhexidine gluconate mouthwash groups. However, in the acute phase, the myrrh mouthwash showed a positive impact on the process of wound healing after implant placement. The small sample size and inability to compare wound healing in different anatomical areas of the oral cavity were the study limitations.

## 1. Introduction

A loss of natural teeth affects routine activities such as speech and eating [1]. The absence of teeth from the dental arch is a routinely observed condition, which may be congenital or caused by diseases such as caries or periodontal breakdown [2]. According to the American Association of Oral and Maxillofacial Surgeons, 69% of adults between the ages of 35 and 44 have lost at least one permanent tooth due to accident, periodontal disease, unsuccessful root canal therapy, or tooth decay [3]. Gaviria et al., 2014 stated that 26% of adults face the loss of approximately all permanent teeth by the age of 74 [3].

A dental implant is a good restorative alternative with long-term results in the rehabilitation of partially or completely edentulous patients [4]. One of the major advantages of placing implants is their attachment to the alveolar bone, which provides strong support for artificial teeth and eliminates the need to prepare the adjacent teeth for supporting the prosthesis [5].

Maintaining good oral hygiene is essential for any treatment procedure. Studies have suggested that the mean brushing time is less than the time required for proper cleaning. Moreover, only 2–10% of patients use dental floss regularly and effectively [6,7]. Despite educating and motivating patients to use toothbrushes and floss properly, compliance is reduced over time. Special motivational skills are needed to educate people on the optimal use of toothbrushes and floss [8]. Inadequate practices of dental hygiene result in the accumulation of plaque, mostly on the interproximal surfaces of teeth. Under such conditions, mouthwash becomes important in dentistry because it contains various antimicrobial agents to complement the expected outcomes of mechanical oral hygiene measures [9].

The efficacy of antiseptic mouthwashes containing chlorhexidine (CHX) and essential oils (EO) as active ingredients to control plaque formation and gingivitis has been demonstrated in previous studies [10,11]. Microbial organisms have been successfully reduced through the use of antimicrobial mouthwash, which is held in the mouth and swished by the action of the perioral musculature to eliminate the oral pathogens [12,13]. The daily use of mouthwash is recommended for proper oral hygiene. Some mouthwashes are based on natural substances; myrrh is one of them because it is an oleo-gum resin, which is a natural substance obtained from the herbs Commiphora molomol and Balsamodendron myrrh [14]. It comprises volatile oil (7–17%), resin (25–40%), gum (57–61%), and impurities (3–4%) [14,15,16,17,18].

CHX is a broad-spectrum, antimicrobial, and antifungal agent that belongs to a class of drugs named biguanides [19]. It potentiates wound healing and decreases postoperative problems [20]. The use of myrrh is common in Saudi Arabia and the Middle East and there is published research that highlights the favorable results of using myrrh [14,21]. For many years, myrrh has been in use for the healing of wounds [21]. Both CHX and myrrh have shown beneficial effects in wound healing. To the best of our knowledge, there are no studies comparing the effects of these mouthwashes after implant placement on soft tissue healing. Therefore, the objective of the study was to determine the efficacy of myrrh and CHX mouthwashes on soft tissue healing after implant placement.

## 2. Materials and Methods

### 2.1. Study Design

This prospective double-blinded clinical trial was conducted according to the Institutional Review Board (IRB) principles in Saudi Arabia. All patients were informed about the study protocol and signed informed consent forms were obtained. The research Ethics Committee (REC) of King Fahd Central Hospital, Jazan, Saudi Arabia, approved the study protocol.

### 2.2. Study Population

**Figure 1:**
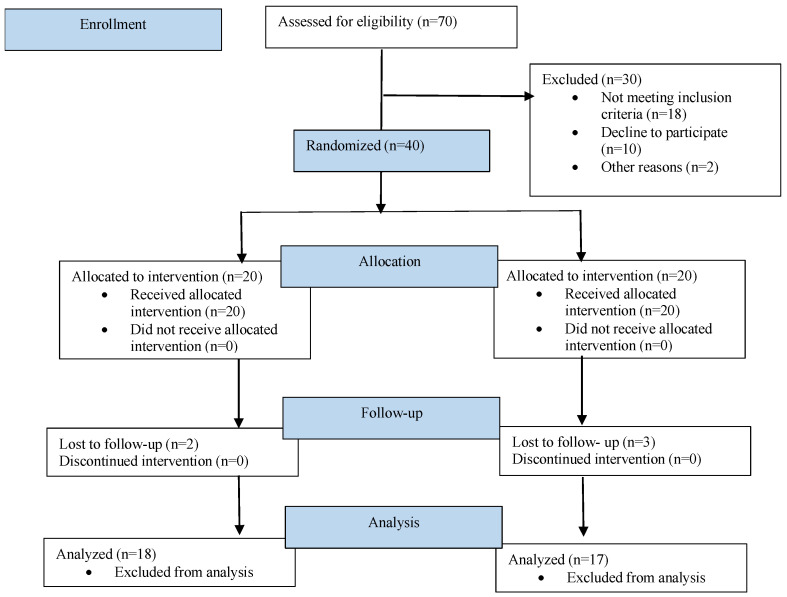
Flow diagram of study procedures.

A total of 35 patients were included in the study according to the inclusion and exclusion criteria. The inclusion criteria for patients included healthy individuals aged between 20 and 50 years of both genders, with good oral hygiene with a full mouth plaque score of less than 1, bleeding on probing less than 10%, and an indication of a two-stage implant placement procedure. Patients were excluded under the following conditions: a plaque score greater than 1, bleeding on probing greater than 10%, pregnancy, chronic smoking, full mouth rehabilitation, a history of head and neck region radio- or chemotherapy for the past two years, and uncontrolled diabetes and hypertension. A flow diagram of the study procedures is shown in Figure 1.

Patients were divided into two groups using simple randomization (without stratification) by randomly allocating odd or even numbers and then the patients were grouped into A and B groups. The participants and principal investigator were double-blinded to the patients’ randomization, which was reported by a neutral dental assistant. Group A (chlorhexidine group) contained 17 patients and Group B (myrrh group) contained 18 patients. The sample size determinations were based on a review of the previous literature, where similar trials with a focus on the effects of mouthwash were conducted [22,23].

After providing instructions about oral hygiene, the external operator (author) randomly distributed the assigned product to the patients. Each mouthwash was labeled with a code to reduce bias. The products were kept in a colored bottle to avoid any impairments caused by sunlight and prevent participants from identifying the products. Patients were distributed into two groups:

Group A: Patients were given a CHX 0.12% (Kin gingival complex, Laboratories KIN) mouthwash, twice a day for two weeks.

Group B: Patients were given a myrrh mouthwash twice a day for two weeks.

The following instructions for using the mouthwash were provided: 30 min after brushing, rinsing with the mouthwash was advised for 30 s, with no eating or drinking for 30 min after use.

Patients were advised not to modify their usual oral hygiene maneuvers during the study period and to strictly follow the instructions. All patients were prescribed Amoxicillin 500 mg TID for one week. Patients who were allergic to Amoxicillin were prescribed Clindamycin 300 mg TID for seven days.

### 2.3. Preparation of Myrrh Mouthwash

A 1% myrrh mouthwash was prepared as per Bassiouny G, Barrak [24]. An amount of 50 gm of myrrh (the oleogum resin obtained from the stems and branches of Commiphora molmol) was washed with cold water and dried. It was infused in 5 L of warm water (45 °C) for 24 h followed by ultra-sonic shaking for four hours [24]. The solution was left overnight, then filtered and stored in a 250 mL sealed bottle before use.

### 2.4. Assessment of Soft Tissue Healing

The postoperative soft tissue healing assessment was carried out by a clinical assessment, an assessment of postoperative patient responses, and a histopathological examination.

#### 2.4.1. Clinical Assessment

The postoperative assessment was carried out clinically by determining the wound opening and the color and swelling of the tissue.

The gingiva swelling was scored as follows: 0 = no swelling and 1 = swelling presented. The gingival color was recorded as follows: 0 = no redness and 1 = redness presented. The contralateral gingiva was used as a reference for evaluating the modifications in the tissue color. The presence or absence of facial asymmetry or swelling was evaluated to obtain local findings, which were reported by visually comparing the implant site with the contralateral side and measuring between facial points made using permanent markers before the surgery. Differences in the distances between the preoperative and postoperative marks were used as the basis for the calculations.

#### 2.4.2. Patient Feedback Questionnaire

At one week postoperative, patients’ feedback was evaluated using a questionnaire to record their experiences with the mouthwash and determine postoperative problems. The study questionnaire consisted of 5 questions. The first two questions were about the mouthwash (proper use and satisfaction). The remaining three questions were about postoperative problems.

#### 2.4.3. Histological Examinations

**Figure 2:**
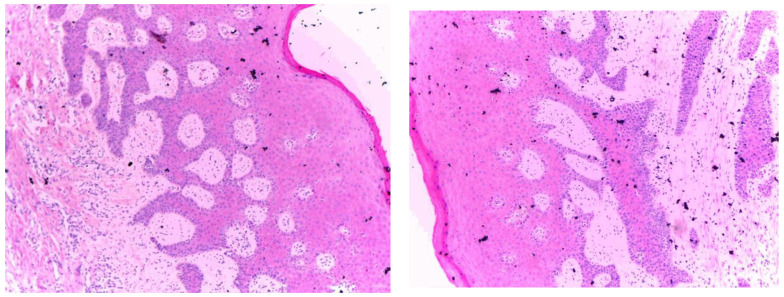
Histopathological examination of the myrrh group.

**Figure 3:**
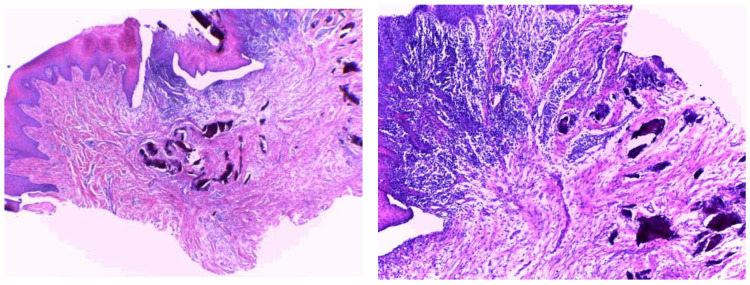
Histopathological examination of the CHX group.

After two months, the Modified Connective Tissue Punch (MCTP) technique was used for the second stage to obtain the tissues after evaluating Keratinized Tissue Width (KDW). Only one incision was made using a motor-driven circular tissue punch (FAL-31-006-010, FMD, Rome, Italy) of the same diameter as the prosthetic platform of the selected implant. This first incision marked the profile of the connective punch. An adequate KDW is required to prevent the implant from recessing. A sample from each group (only three patients from each group) was sent for the histopathological examination at King Fahd Central Hospital to evaluate the infiltration of inflammatory cells (using inflammatory extent and inflammatory cellularity grades), maturation of collagen (fibroblast activity), and arrangement of cells (for detecting the remodeling phase). Figure 2 and Figure 3 show samples from patients from the myrrh and CHX groups, respectively.

### 2.5. Statistical Analysis

Data obtained were analyzed using the Chi-square test for redness and swelling, whereas the Mann–Whitney U-test was used for the wound healing differences between the groups. The Statistical Package for Social Sciences (SPSS) version 25.0 program was used (SPSS Inc., Armonk, NY, USA). The variables were presented using descriptive statistical analysis including frequencies, mean percentages, and standard deviations.

## 3. Results

### 3.1. Study Population

Thirty-five patients participated in the study. Participants were divided into two groups: Group A (chlorhexidine group) who received the 0.12% CHX mouthwash and Group B (myrrh group) who received the 1%myrrh mouthwash.

In the CHX group, 52.29% were male and 47.05% were female. In the myrrh group, 33.3% were male and 66.7% were female. The demographic data of the group are shown in Table 1.

### 3.2. Tissue Healing Assessment

#### 3.2.1. Patient Feedback Questionnaire

All the patients responded to all the questions in the feedback questionnaire. The questions and responses are shown in Table 2. Patients were more compliant in the myrrh group with the use of mouthwash. All (100%) of the patients in the myrrh group used the mouthwash as per the instructions, whereas 94.1% of the patients in the CHX group used the mouthwash as per the instructions. The participants in both groups were satisfied with the mouthwash they used. The CHX group showed higher satisfaction than the myrrh group but the results were not statistically significant.

In the CHX group, 76.4% of participants responded that bleeding stopped one hour after surgery compared to 66.7% in the myrrh group. The results were not statistically significant. Less sensitivity (16.7%) was reported by patients in the myrrh group compared to the CHX group (41.1%). Fewer patients (22.2%) in the myrrh group reported pain one day postoperatively compared to 35.29% of patients in the CHX group.

#### 3.2.2. Clinical Assessment

In the myrrh group, 94.4% of patients had no swelling and only 5.6% of patients had swelling. No swelling was seen in the CHX group but the difference was not statistically significant. In the myrrh group, 16.7% of participants reported redness, whereas none of the participants in the CHX group showed any signs of redness (Table 3). There were no complaints reported by patients in both groups regarding wound opening and no statistically significant between-group difference was reported.

#### 3.2.3. Histopathological Results

Figure 2 shows the histopathological results of the myrrh group. Histopathologically, well-oriented, stratified, squamous epithelium with elongated rete ridges can be seen. The stratifications are well maintained. The underlying connective tissue stroma shows regular mature bundles of collagen fibers with mild chronic inflammatory cell infiltration. No signs of dysplasia or malignancy can be seen.

Figure 3 shows the histopathological results of the CHX group. Histopathologically, well-oriented, stratified, squamous epithelium with elongated rete ridges can be seen. The underlying connective tissue stroma shows irregular bundles of collagen fibers with moderate inflammatory cell infiltration. There are no signs of dysplasia or malignancy.

In comparison with the CHX group, the myrrh group has dense collagen fibers, whereas regular and mild inflammatory cell infiltration is seen in the CHX tissue sections, which is suggestive of better healing properties.

## 4. Discussion

Wound healing is a dynamic process in tissue injuries and involves the reinstatement of cellular and tissue structures. Various chemical agents have been used to enhance wound healing in both non-surgical and surgical therapies. This study evaluated the effects of myrrh and CHX mouthwashes in postimplant patients. It was found that most of the patients in the myrrh and CHX groups did not report any considerable postoperative impairments, infections, or symptoms throughout the postoperative evaluation. However, a few patients in the myrrh group reported redness. Less sensitivity and pain after surgery were reported in the myrrh group.

In an extraction wound study, Rania Eid found improved wound healing one week postoperatively with a myrrh mouthwash and 90% of participants showed reduced socket opening with a myrrh mouthwash compared to 40% of participants in the control group. A significant decrease in inflammatory signs was found in the myrrh group compared to the control group [25]. In our study, improvements in wound healing were seen with both the myrrh and CHX mouthwashes. In contrast to our study, Alotaibi et al., 2020 showed a higher reduction of gingival inflammation in the CHX group compared to the myrrh group [26].

In an animal study, Al-Mobeeriek found better results with a myrrh mouthwash after comparing 0.2% myrrh, 0.2% chlorhexidine gluconate, and 0.25% tetracycline mouthwashes with a saline mouthwash [27]. The myrrh group showed more remodeling in the early stages compared to the other mouthwash groups. In a double-blind study, Zahid et al., showed improved wound healing with a myrrh mouthwash [28]. In that study, they evaluated the efficacy of a 1%myrrh mouthwash and a 0.2% chlorhexidine mouthwash and found that myrrh and CHX showed a reduction in gingival inflammation but the difference was not statistically significant [28]. This is in agreement with our study, which also showed a reduction in inflammation, wound opening, and swelling in both groups, but these were not statistically significant. Our results are also in agreement with Bassiouny and Barrak who demonstrated a reduction in gingival inflammation with a myrrh mouthwash [24]. Comparable results of myrrh and CHX mouthwashes were also reported by Sambawa et al. in terms of antibacterial activity [29]. However, Almeklafi found higher antibacterial activity in the CHX group than in the myrrh group [30].

No side effects, signs of toxicity, allergy, or unexplained impairment were reported using the myrrh mouthwash. This is due to the low dose, high-tissue tolerance, and short duration of the application of myrrh [31]. These findings are corroborated by previous findings regarding the efficacy of myrrh in reducing tissue toxicity using different mechanisms. This could be because of its stimulatory impact on mucous secretion as well as its antioxidant, immune, and antitumor properties [32,33]. Myrrh also enhances leukocyte migration to the injury site and helps to maintain their function and accelerate the healing process [34]. Myrrh also exhibits an inhibitory action on inflammatory mediators such as prostaglandins, nitric oxide, and tumor necrosis factor-E2 [35]. This inhibitory action results in a reduction of pain. In an animal study, Shalaby demonstrated that myrrh has analgesic effects, resulting in a reduction of pain [34]. Similar findings were found in our study with a smaller number of patients reporting pain one day postoperatively in the myrrh group compared to the CHX group. The histological findings of our study showed well-oriented epithelium, dense and regular collagen fibers, and mild inflammatory cell infiltration in the myrrh group compared to the CHX group. This is suggestive of better wound healing in the myrrh group. However, the difference was not significant. These findings suggest that myrrh plays a role in wound healing. Improved healing results in a reduction of wound morbidity and can reduce the expense of the treatments occurring as a result of postoperative complications [36]. Taheri et al. also highlighted the soothing effects of myrrh on inflamed tissues in oral cavities and the throat when used as a mouthwash [37]. To the best of our knowledge, this is the first study that involves an evaluation of the wound-healing properties of myrrh mouthwash in comparison with CHX mouthwash during the postimplant period. However, further studies with complicated wounds and increased sample sizes are required.

## 5. Conclusions

The results of the present study in combination with the available scientific literature provide evidence that myrrh mouthwash could be used as an adjunctive therapeutic agent in post-implant therapy.

## Figures and Tables

**Table 1:** Demographic data of study population.

Characteristics	Myrrh Group (*n* = 18)	CHX Group (*n* = 17)
Male	6 (33.3)	9 (52.29)
Female	12 (66.7)	8 (47.05)
Age	31.78 ± 6.89	34.52 ± 8.23

**Table 2:** Patient feedback questionnaire: questions and responses.

Questions	Myrrh Group (*n* = 18)	CHX Group (*n* = 17)	*p*-Value
1. Did you use the mouthwash frequently as instructed?	Yes 18 (100)	16 (94.1)	0.332
2. What is your satisfaction with this mouthwash after implant placement?	9.38 ± 0.69	9.47 ± 0.62	0.579
3. When did bleeding stop at the surgical site? One hour after surgery Immediately after surgery 2nd day after the procedure	12 (66.7) 3 (16.7) 3 (16.7)	13 (76.4) 1 (5.8) 3 (17.6)	0.668
4. Did you experience any sensitivity at the surgical site after using the mouthwash? Yes No	3 (16.7) 15 (83.3)	7 (41.1) 10 (58.9)	0.096
5. For how long did you experience pain after the procedure? One day Two days Three days One week	4 (22.2) 6 (33.3) 5 (27.8) 3 (16.7)	6 (35.29) 2 (11.7) 3 (17.6) 6 (35.2)	0.653

**Table 3:** Clinical assessment.

Variables	Myrrh Group	CHX Group	*p*-Value
Wound opening Swelling Absent Present	0.33 ± 0.52	0.32 ± 0.30	0.945
	17 (94.4) 1 (5.6)	17 (100) 0	0.032
Redness around the surgical site Absent Present			
	15 (83.3) 3 (16.7)	17 (100) 0	0.0632

## Data Availability

Data are included in the study.
